# Alexander Bailey

**Published:** 1917-03

**Authors:** 


### OBITUARY

**Alexander Bailey,** M. D., died Dec. 24, near Morocco, Ind., aged 96.

